# Cytomegalovirus-associated encephalomyelitis in an immunocompetent adult: a two-stage attack of direct viral and delayed immune-mediated invasions. case report

**DOI:** 10.1186/s12883-016-0761-6

**Published:** 2016-11-17

**Authors:** Kensuke Daida, Yuta Ishiguro, Hiroto Eguchi, Yutaka Machida, Nobutaka Hattori, Hideto Miwa

**Affiliations:** 1 Department of Neurology, Juntendo University Nerima Hospital, 3-1-10 Takanodai, Nerima, Tokyo, 177-8521 Japan; 2 Department of Neurology, Juntendo University School of Medicine, 1-21-1 Hongo, Bunkyo, Tokyo, 113-0033 Japan

**Keywords:** Cytomegalovirus, Transverse myelitis, Acute disseminated encephalomyelitis, Immunocompeten, Case report

## Abstract

**Background:**

It is clinically rare to find cytomegalovirus (CMV)-associated encephalomyelitis in immunocompetent adults. Here, we present the case of an adult patient who developed acute transverse myelitis that was followed by immune-mediated disseminated encephalomyelitis.

**Case presentation:**

A 38-year-old man developed acute paraplegia with paresthesia below the level of the T7-8 dermatome. Both brain and spinal cord MRIs performed at admission appeared normal. Corticosteroid therapy was initiated, with the later addition of high-dose intravenous immunoglobulins. After polymerase chain reaction analysis indicated the presence of CMV DNA in his cerebrospinal fluid (CSF), anti-viral therapy was added. Forty days after symptom onset, despite an initial positive response to this therapy, he developed dysarthria and truncal ataxia. Repeated magnetic resonance imaging scans demonstrated progressively expanding lesions involving not only the spinal cord but also the cerebral white matter, suggestive of extensive immune-mediated demyelination involving the central nervous system (CNS), as is observed in acute disseminated encephalomyelitis (ADEM).

**Conclusion:**

This case report underscores the importance of careful patient observation following the initial diagnosis of a CMV-associated CNS infection, such as transverse myelitis, on the possibility that post-infectious ADEM may appear.

## Background

Cytomegalovirus (CMV) is one of herpes viruses, and it infects only humans. It is well known that CMV causes central nervous system (CNS) infections in immunocompromised patients, such as in patients with human immunodeficiency virus (HIV) infection or in organ transplant recipients. By contrast, CMV infection is typically subclinical in healthy adults. However, a few reports have described CMV encephalomyelitis occurring in immunocompetent patients [1–12]. Recently, we encountered a patient with CMV-associated acute transverse myelitis who developed extensive demyelinating lesions involving the CNS, similar to those observed in acute disseminated encephalomyelitis (ADEM), after an interval of 40 days.

## Case presentation

**Fig. 1:**
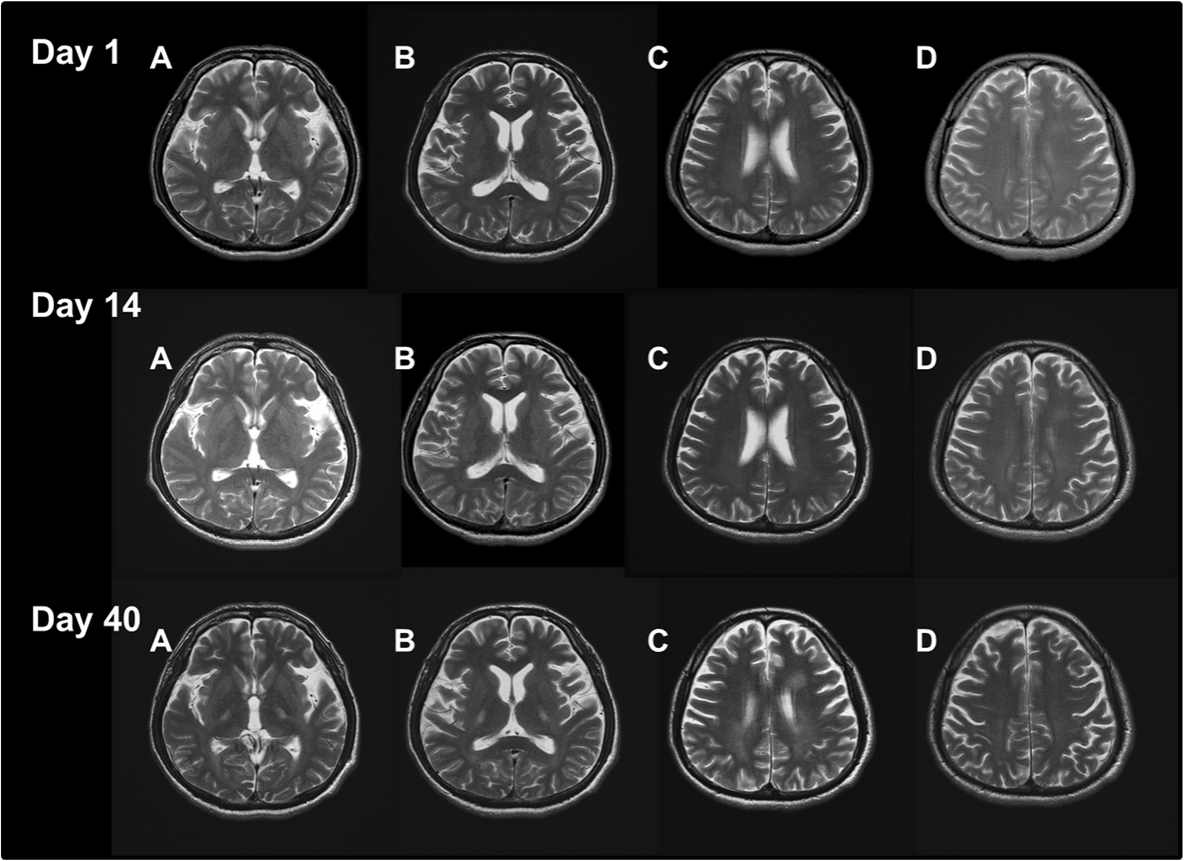
T2-weighted brain MR images on day 1, 14, and 40 after admission. In MR images on day 40, hyperintense foci on T2-weighted images are unequivocally demonstrated in the bilateral internal capsules (Day 40, a and b) and cerebral white matter near the lateral ventricles (Day 40, c)

**Fig. 2:**
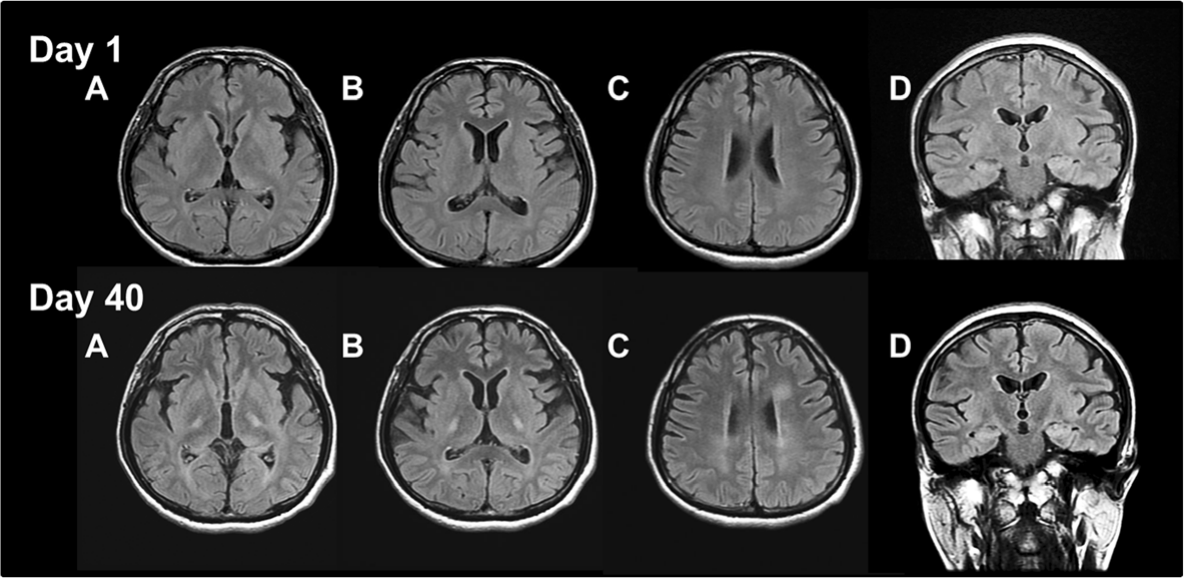
FLAIR MR images of the brain on day 1 and day 40 after admission. The images on day 40 show that hyperintense foci are unequivocally present in the bilateral internal capsules (Day 40, a, b and d), periependymal regions of the posterior horns of lateral ventricles (Day 40, a, b and d), and cerebral white matter near the lateral ventricles (Day 40, c)

**Fig. 3:**
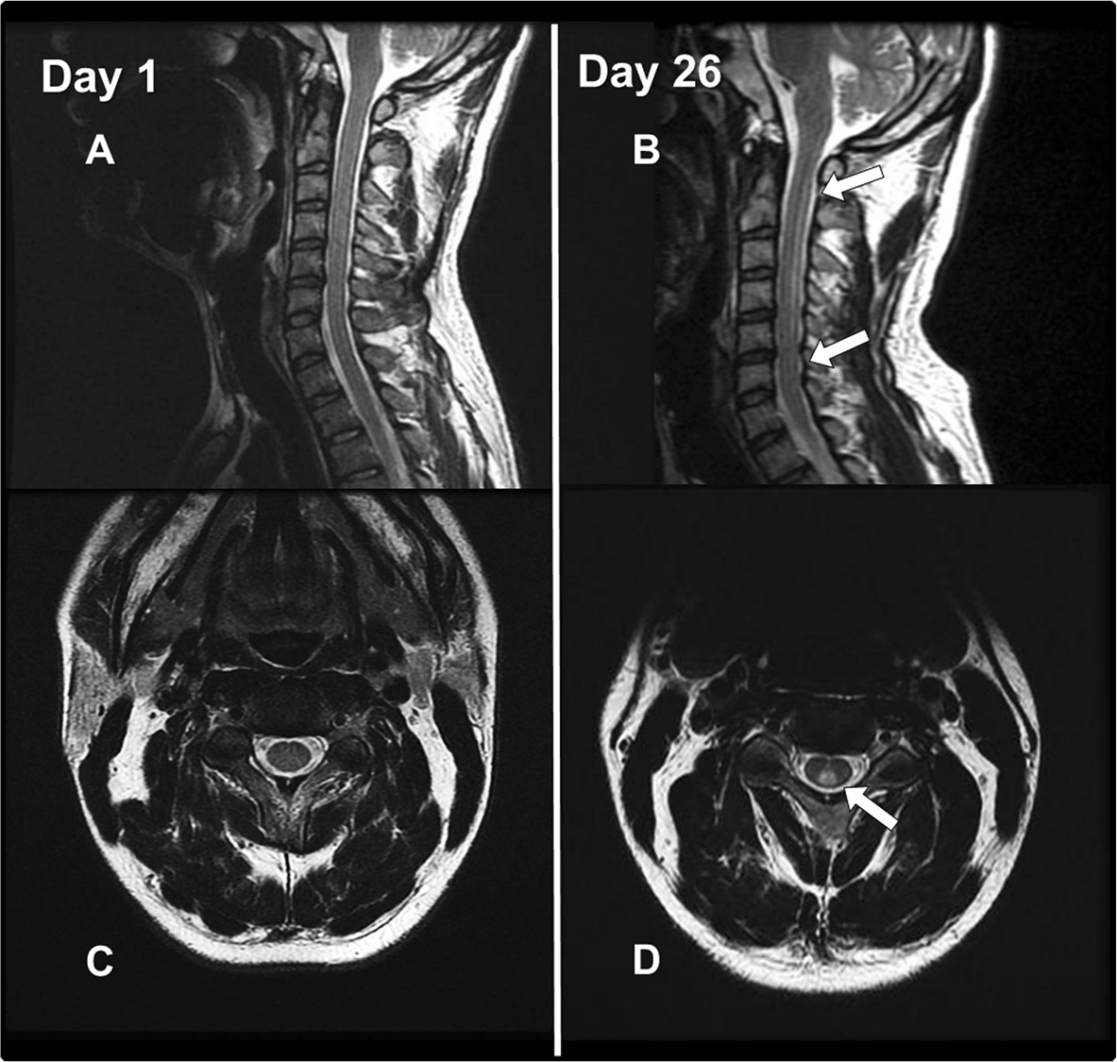
T2-weighted MR images of the spinal cord on day 1 and day 26 after admission. On day 26, hyperintense lesions are present at cervical C2–C6 levels (B, *arrows*). White matter hyperintensities are also observed on T2-weighted MR images in the posterior portion of the spinal cord (d)

**Fig. 4:**
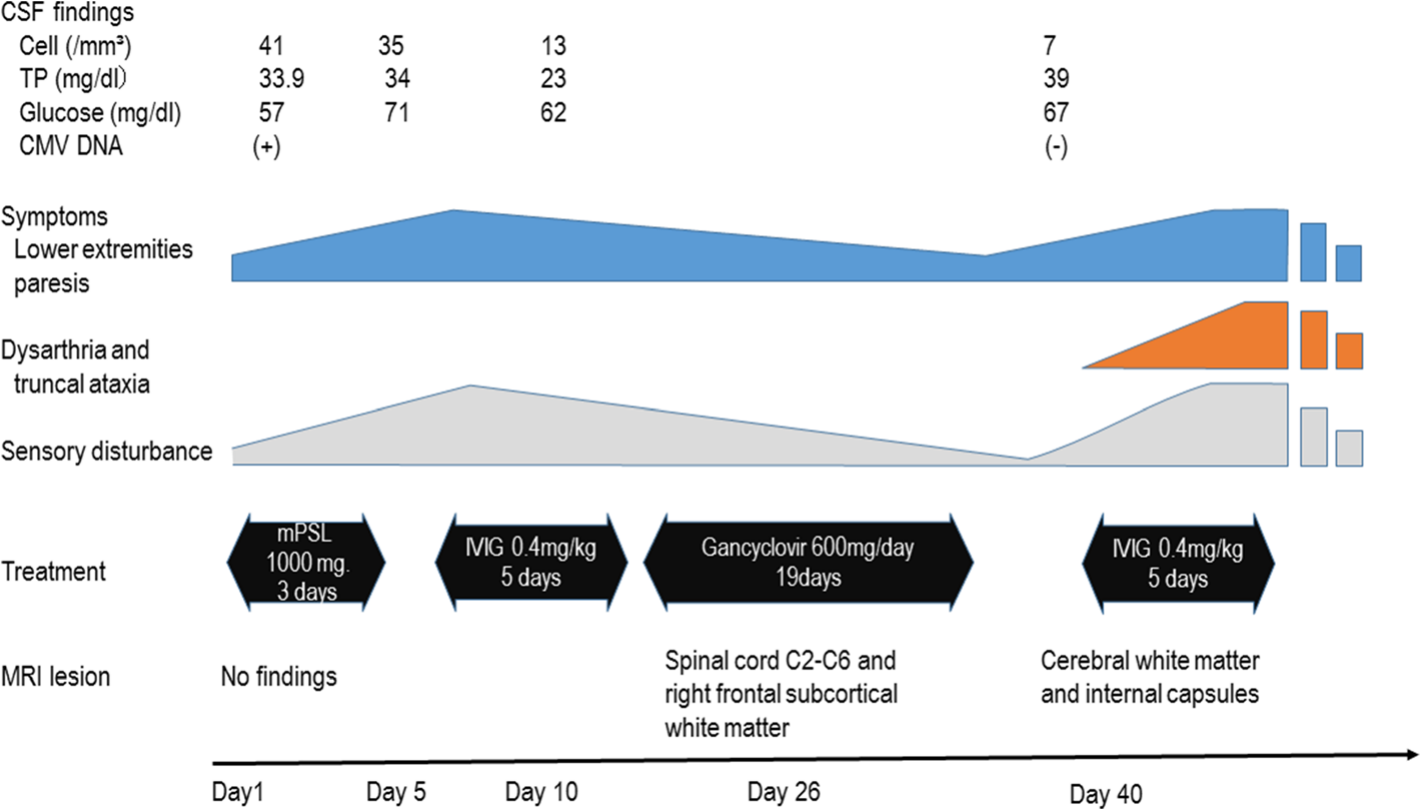
Time course of clinical symptoms, CSF results, treatment, and MRI findings

A 38-year-old Japanese man was admitted to our hospital because of muscle weakness in his lower extremities. His and his family’s histories were unremarkable. Fifteen days before admission, he had a mild fever with fatigue. Simultaneously, itchy skin rashes emerge on his foot and face, particularly around his mouth. The patient was tentatively diagnosed as having hand, foot, and mouth disease. Prior to admission, his fever, fatigue, and skin rash began to resolve, but the lower limb weakness progressively worsened within a few days. On admission, his general condition was unremarkable. A neurological examination showed that he was alert and oriented. His higher cerebral functions and cranial nerves were intact. The patient showed spastic paraparesis, with weakness of both lower extremities at approximately 4/5 strength. Deep tendon reflexes were brisk in all extremities, with ankle clonus in both legs. Babinski sings were bilaterally positive. He had paresthesia below the level of the T7-8 dermatome. Difficulty in micturition was noted. The patient had no sign of meningeal irritation. The results of his laboratory tests showed that his complete blood cell count, chemistry, immunoglobulin levels, C-reactive protein, erythrocyte sedimentation rate, and urinalysis were all within reference values. In particular, alterations in the liver function test results, suggestive of infectious mononucleosis, were not observed. Serological tests for syphilis, hepatitis B and C, HIV, and human T-lymphotropic virus type 1 were negative. The test results were also negative for anti-nuclear antibodies, anti-double stranded DNA, and cytoplasmic and perinuclear types of antineutrophil cytoplasmic antibodies, antiphospholipid antibodies, and anti-aquaporin-4 antibody. Antibody titers were not elevated for herpes simplex virus immunoglobulin M (IgM), varicella zoster virus IgM, Epstein–Barr virus IgM, and CMV IgM. CMV IgG was found elevated significantly. Additionally, the tests for coxsackie A16 and enterovirus were not significantly elevated, although we could not perform a serodiagnosis with paired serum samples. A malignancy survey, in which contrast-enhanced CTs of the chest, abdomen, and pelvis were included, was conducted in the present patient, and no cancer was identified. Additionally, tumor markers (alpha-fetoprotein, CEA, CA19-9, and soluble interleukin-2 antigen) were all within normal ranges. Examination of CSF showed elevated white blood cells, although protein (34 mg/dl) and glucose (57 mg/dl) levels were within normal ranges. The CSF IgG index 0.8 was found to be mildly elevated. The myelin basic protein (40.0 pg/ml) levels were not increased, and there were no oligoclonal IgG bands in the CSF sample. Nerve conduction velocity studies of the peripheral nerves indicated that they were intact. Sensory evoked potentials obtained by tibial nerve stimulation demonstrated no reproducible waves. Both brain and spinal cord magnetic resonance images (MRI) obtained at admission appeared normal (Figs. 1, 2, and 3). Brain and spinal cord MRI examinations with gadolinium-enhancement were also performed, although no significant enhancement was demonstrated. After admission, the patient’s weakness and deep sensation disturbance of the lower extremities progressively worsened. He was tentatively diagnosed with transverse myelitis, and treatment was started with intravenous methylprednisolone at a dose of 1000 mg/day for 3 days, followed by oral prednisolone (PSL) (60 mg/day). After 7 days, the muscle weakness in his lower extremities continued to worsen, and we added intravenous immunoglobulin therapy (IVIG) at a dose of 0.4 mg/kg for 5 days. Ten days after admission, intravenous administration of ganciclovir (600 mg/day, 19 days) was initiated because CMV DNA was found in his CSF following polymerase chain reaction analysis (PCR) performed at admission. The deterioration of his symptoms ceased, and the weakness in his lower extremities gradually recovered. Other findings in his CSF also improved, including the results for white blood cells (13/mm^3^; all cells were lymphocytes), protein (23 mg/dL), and glucose (62 mg/dL). The CSF samples were also negative for CMV. However, MRI performed again on day 26 after admission demonstrated a spinal cord lesion of high signal intensity at cervical levels C2–C6, suggesting that the spinal cord was involved and may have been responsible for the paraplegia, although his paraplegia continued to improve with treatment (Fig. 3). Additionally, a hyperintense signal was observed in the right frontal subcortical white matter (Figs. 1 and 2). On day 40 after admission, the weakness and numbness of his lower extremities again worsened, and myoclonic movements appeared in his lower legs. Subsequently, a loss of finger dexterity appeared bilaterally. In addition, the patient developed dysarthria and truncal ataxia, the latter of which resulted in his inability to maintain a sitting position. Brain MR images demonstrated faint, irregular-shaped lesions involving cerebral white matter with dense T2-weighted and fluid attenuated inversion recovery (FLAIR) hyperintensities in the internal capsules, suggesting that extensive disseminated demyelination was actively ongoing (Figs. 1 and 2). The CSF examination results showed re-elevation of protein (39 mg/dL), and MBP (58.9 pg/mL) was also increased compared with results from the initial CSF study, although both levels were within normal ranges. The white blood cell count in the CSF did not increase (7/mm^3^). We considered the possibility that the patient had ADEM, and intravenous methylprednisolone (1000 mg/day, 3 days) was administered again, followed by IVIG (0.4 mg/kg, 5 days). After this treatment, his neurological deterioration ceased, but he had residual neurological sequelae. Time course of clinical symptoms, CSF results, treatment, and MRI findings in the present patient are schematically shown (Fig. 4).

## Discussion

The signs and symptoms in the present patient were manifested as a biphasic clinical course. The patient’s illness started as acute transverse myelitis, but during his recovery, he had extensive demyelination of the cerebral white matter. There was a time lag of about 40 days between the two episodes. We speculated that different pathophysiologies were responsible for this two-stage attack: the first attack was caused by direct viral invasion, and the second by an indirect, immune-mediated mechanism.

Based on the PCR results, we concluded that the acute transverse myelitis was caused by direct invasion of CMV, in particular by a reinfection, because CMV IgM but not IgG was not elevated in his CSF. However, it is rare to detect CMV DNA in the CSF obtained from patients with CMV-associated transverse myelitis. To date, 12 immunocompetent patients with CMV-associated transverse myelitis have been reported [1–12]. In only one of these patients was CMV DNA detected by PCR in the CSF. However, none of these 12 patients showed a two-stage attack, as in our case. Recently, Arslan et al. [1] reviewed nine CMV myelitis case reports and found poor prognosis in eight. Symmetrical symptoms with moderate to severe spinal cord dysfunction [1] and rapid progression of symptoms [5] are associated with poor outcomes, but factors related to better outcomes are as yet unclear.

We speculated that the second wave of the two-stage attack in the present patient might have been caused by an immune-mediated mechanism for the following reasons. The neurological and laboratory findings between the first and second attacks were quite different. From a neurological standpoint, the symptoms observed in the first attack suggested acute transverse myelitis. However, the second attack was associated with not only a deterioration of the symptoms of transverse myelitis but also the appearance of new symptoms, including dysarthria, clumsiness of upper extremities, and truncal ataxia, suggesting that the problem might involve the brain beyond the spinal cord. Regarding the laboratory findings, the re-elevation of CSF protein was noted during the second attack, although the extent of the elevation was mild. However, the MRI findings differed markedly between the first and second attacks. The appearance of multiple disseminated white matter lesions with hyperintense signals in the internal capsules during the second attack supported the speculation that extensive demyelination was occurring, as in ADEM. It has been previously hypothesized that CMV potentially has an antigen-presentation capacity because the human CMV major capsid protein shares sequence similarity with an encephalitogenic myelin/oligodendrocyte glycoprotein (MOG 34–56) [13].

Recently, Tenenbaum and colleagues proposed that biphasic disseminated encephalomyelitis (BDEM) is a variant of ADEM [14]. According to their proposal, patients manifest a biphasic course, with the second attack occurring at least 1 month after the initial event showing different symptoms and radiologic evidence of new lesions at a different site. They reported that approximately 10% of patients with ADEM had the biphasic disease [14]. The clinical course and neuroimaging findings of the present patient may generally meet the proposed criteria for BDEM. Indeed, it was reported that CMV infection is a potential cause of BDEM [15]. However, there are clear differences in the pathophysiological mechanisms observed in BDEM and in the present patient. In BDEM, not only the second but also the first phase of the illness is caused by ADEM. In the present patient, as we speculated, the first phase of illness was caused by direct invasion of CMV. Although it is possible that the present patient might have BDEM, it is also plausible that CMV was coincidentally re-activated during the initial episode of illness.

One additional differential diagnosis that should also be considered is seronegative neuromyelitis optica spectrum disorder (NMOSD), because the spinal cord and the brain were involved in the present patient. In particular, lesions involving the internal capsule and periependymal regions of the posterior horns of lateral ventricles, both of which are known to be typical MRI neuroimaging of NMOSD, were involved [16]. However, we think that the present patient may not have NMOSD, because of the following reasons: 1) anti-AQP-4 IgG was seronegative; 2) a mildly elevated IgG index also supports ADEM, because the IgG index value is typically not elevated in NMOSD; 3) most core clinical features necessary to fulfill NMOSD were lacking, such as optic neuritis, area postrema syndrome (hiccup, nausea and vomiting), narcolepsy or acute diencephalic syndrome, or acute brainstem syndrome. Additionally, there is a clear difference in distribution of spinal cord lesions between NMOSD and the present patient. NMOSD spinal cord lesions are characterized by exclusive involvement of gray matter; whereas the present patient exhibited a lesion in the posterior portion of the spinal cord. However, although the likelihood that he has NMOSD is not great, careful follow-up is necessary.

## Conclusion

CMV potentially involves the CNS in immunocompetent patients, possibly through a viral invasion or by post- or para-infectious immune-mediated demyelination, similar to what occurs in ADEM. It is clinically important to recognize that CMV infection may precipitate a two-stage attack. This case report underscores the importance of careful observation of immunocompetent patients with CMV, watching out for a secondary attack by post-infectious ADEM following the initial CMV-associated inflammation.

## Abbreviations

ADEM: Acute disseminated encephalomyelitis
BDEM: Biphasic disseminated encephalomyelitis
CMV: Cytomegalovirus
CSF: Cerebrospinal fluid
FLAIR: Fluid attenuated inversion recovery
HIV: Human immunodeficiency virus
HTLV-1: Human T-cell leukemia virus type 1
IgM: Immunoglobulin M
IVIG: Intravenous immunoglobulin therapy
MRI: Magnetic resonance imaging
PCR: Polymerase chain reaction
